# Supplementary material for: Alhagi sparsifolia Harbors a Different Root-Associated Mycobiome during Different Development Stages
Source: Microorganisms. 2022 Nov 30;10(12):2376. doi: 10.3390/microorganisms10122376 (PMC9785364; doi:10.3390/microorganisms10122376)
Supplement: Supplementary file 1 [file microorganisms-10-02376-s001.zip › microorganisms-2049335-supplementary.pdf]

Figure legends

Figure S1 The abundance of root-associated fungi taxa of *A. sparsifolia* across compartments and their correlation with environmental factors. **(a)** Average relative abundances of top 10 phyla from different compartments. The asterisk in the legend indicates that the relative abundance of a phylum or class varies significantly across compartments (ANOVA, $P < 0.05$). **(b)** Pearson's correlation between dominant fungal phyla in each compartment and environmental factors. Abbreviations: TN, total nitrogen (g/kg); TP, total phosphorus (g/kg); TK, total potassium (g/kg); AN, available nitrogen (mg/kg); AP, available P (mg/kg); AK, available potassium (mg/kg); EC, electrical conductivity (mS/cm); SWC, soil water content; SOM, soil organic matter (g/kg); SLA, specific leaf area (cm^2/g).

Figure S2 Chao1 of fungal communities among compartments. Different lowercase letters indicate differences between groups based on the ANOVA.

Figure S3 Venn diagrams show the variation partition of main edaphic factors driving community variation. Abbreviations: TP, total phosphorus (g/kg); TK, total potassium (g/kg); AN, available nitrogen (mg/kg); SWC, soil water content; SOM, soil organic matter (g/kg).

FigureS4 The topological attributes of fungal co-occurrence networks among different compartments.

Figure S5 The topological attributes of *A. sparsifolia* root-associated fungal co-occurrence

networks among different host growth times. 30d, 30-day-old; 60d, 60-day-old; 90d, 90-day-old; 2a, 2-year-old; perennial, over 3-year-old.

Figure S6 (a) The functional redundancy of fungal co-occurrence network modules among different host growth times. **(b)** Pearson's correlations between module eigengenes and environmental factors. Abbreviations: TN, total nitrogen (g/kg); TP, total phosphorus (g/kg); TK, total potassium (g/kg); AN, available nitrogen (mg/kg); AP, available P (mg/kg); AK, available potassium (mg/kg); EC, electrical conductivity (mS/cm); SWC, soil water content; SOM, soil organic matter (g/kg); SLA, specific leaf area (cm^2/g). 30d, 30-day-old; 60d, 60-day-old; 90d, 90-day-old; 2a, 2-year-old; perennial, over 3-year-old.

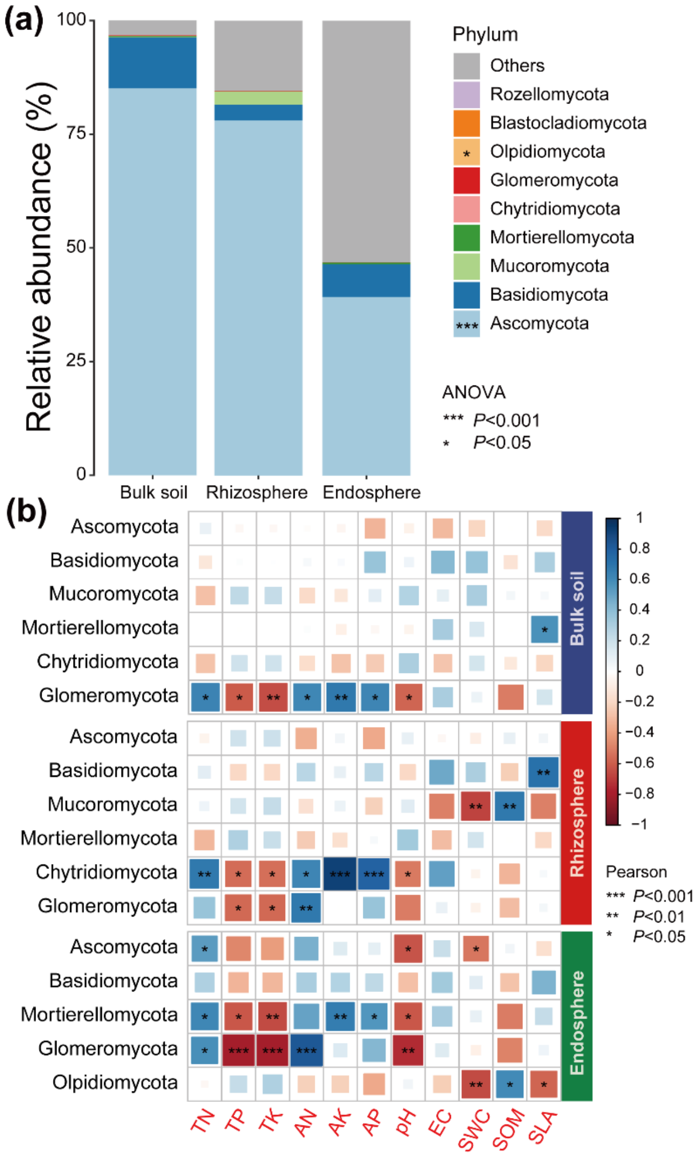
(a)
Relative abundance (%)
100
75
50
25
0
Bulk soil
Rhizosphere
Endosphere
Phylum
Others
Rozellomycota
Blastocladiomycota
* Olpidiomycota
Glomeromycota
Chytridiomycota
Mortierellomycota
Mucoromycota
Basidiomycota
*** Ascomycota
ANOVA
*** P<0.001
* P<0.05
(b)
Bulk soil
Rhizosphere
Endosphere
Ascomycota
Basidiomycota
Mucoromycota
Mortierellomycota
Chytridiomycota
Glomeromycota
Olpidiomycota
TN
TP
TK
AN
AK
AP
pH
EC
SWC
SOM
SLA
1
0.8
0.6
0.4
0.2
0
−0.2
−0.4
−0.6
−0.8
−1
Pearson
*** P<0.001
** P<0.01
* P<0.05

Figure S1

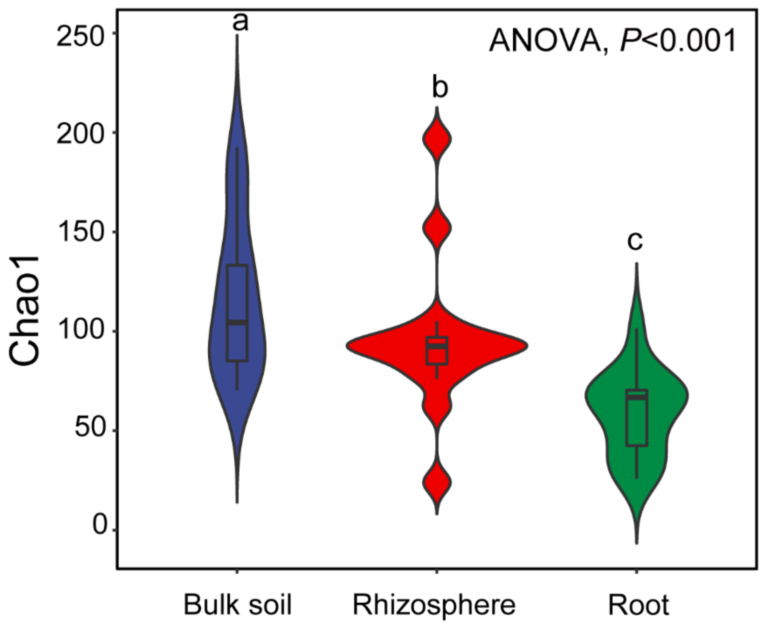

ANOVA, P<0.001
Chao1
250
200
150
100
50
0
a
b
c
Bulk soil
Rhizosphere
Root

Figure S2

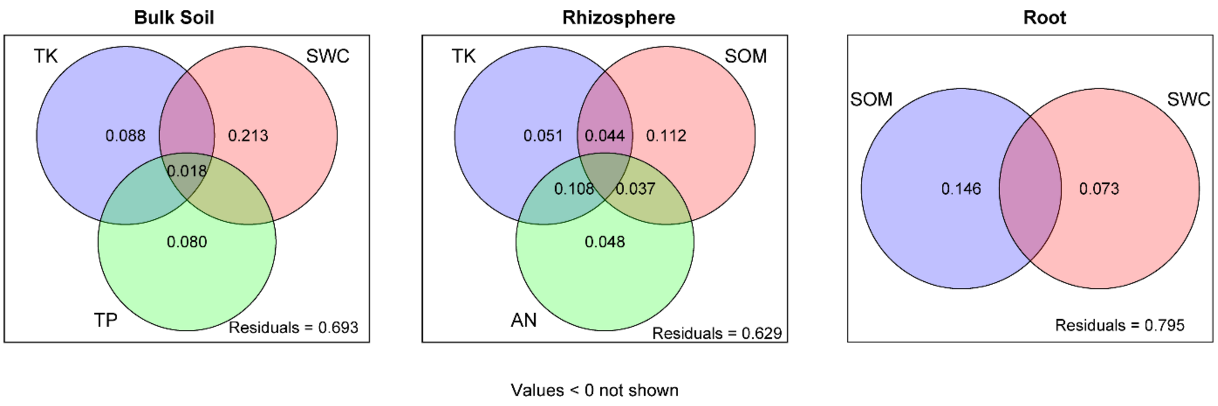
Bulk Soil
TK
SWC
0.088
0.213
0.018
0.080
TP
Residuals = 0.693
Rhizosphere
TK
SOM
0.051
0.044
0.112
0.108
0.037
0.048
AN
Residuals = 0.629
Root
SOM
SWC
0.146
0.073
Residuals = 0.795
Values < 0 not shown

Figure S3

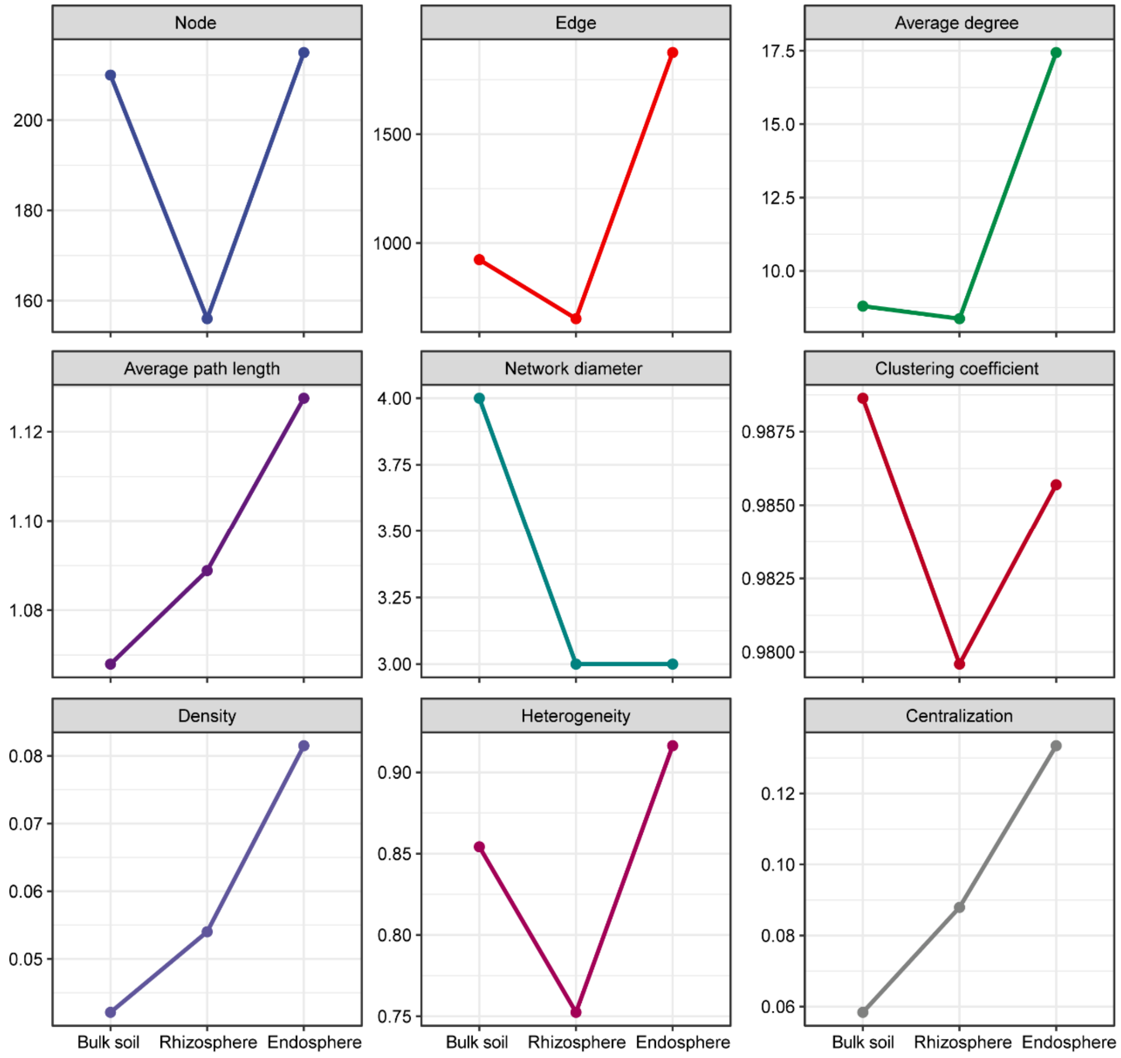
Node
200
180
160
Edge
1500
1000
Average degree
17.5
15.0
12.5
10.0
Average path length
1.12
1.10
1.08
Network diameter
4.00
3.75
3.50
3.25
3.00
Clustering coefficient
0.9875
0.9850
0.9825
0.9800
Density
0.08
0.07
0.06
0.05
Heterogeneity
0.90
0.85
0.80
0.75
Centralization
0.12
0.10
0.08
0.06
Bulk soil
Rhizosphere
Endosphere

FigureS4

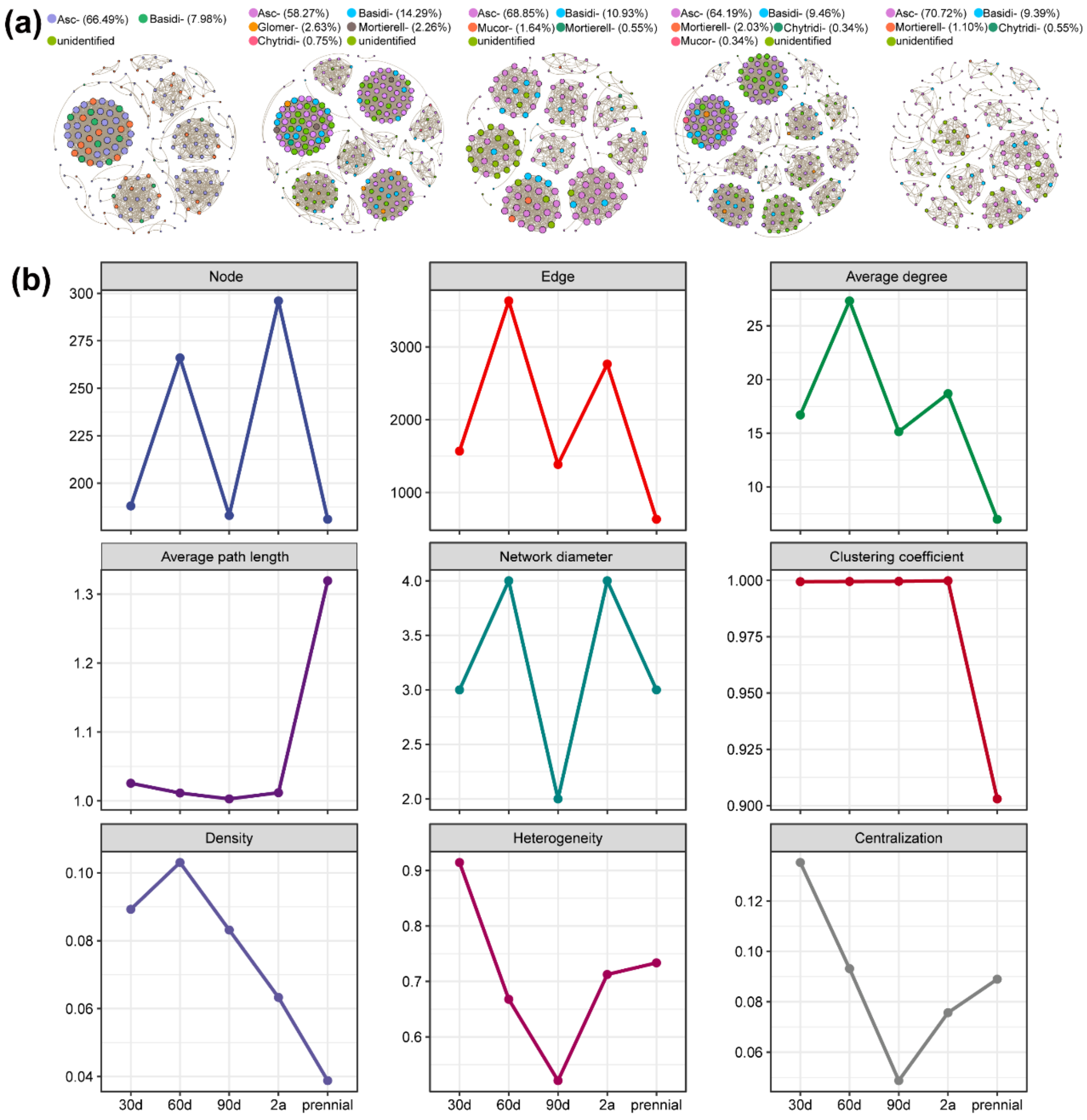
(a)
Asc- (66.49%) Basidi- (7.98%) unidentified
Asc- (58.27%) Basidi- (14.29%) Glomer- (2.63%) Mortierell- (2.26%) Chytridi- (0.75%) unidentified
Asc- (68.85%) Basidi- (10.93%) Mucor- (1.64%) Mortierell- (0.55%) unidentified
Asc- (64.19%) Basidi- (9.46%) Mortierell- (2.03%) Chytridi- (0.34%) Mucor- (0.34%) unidentified
Asc- (70.72%) Basidi- (9.39%) Mortierell- (1.10%) Chytridi- (0.55%) unidentified
(b)
Node
Edge
Average degree
Average path length
Network diameter
Clustering coefficient
Density
Heterogeneity
Centralization
30d
60d
90d
2a
prennial

Figure S5

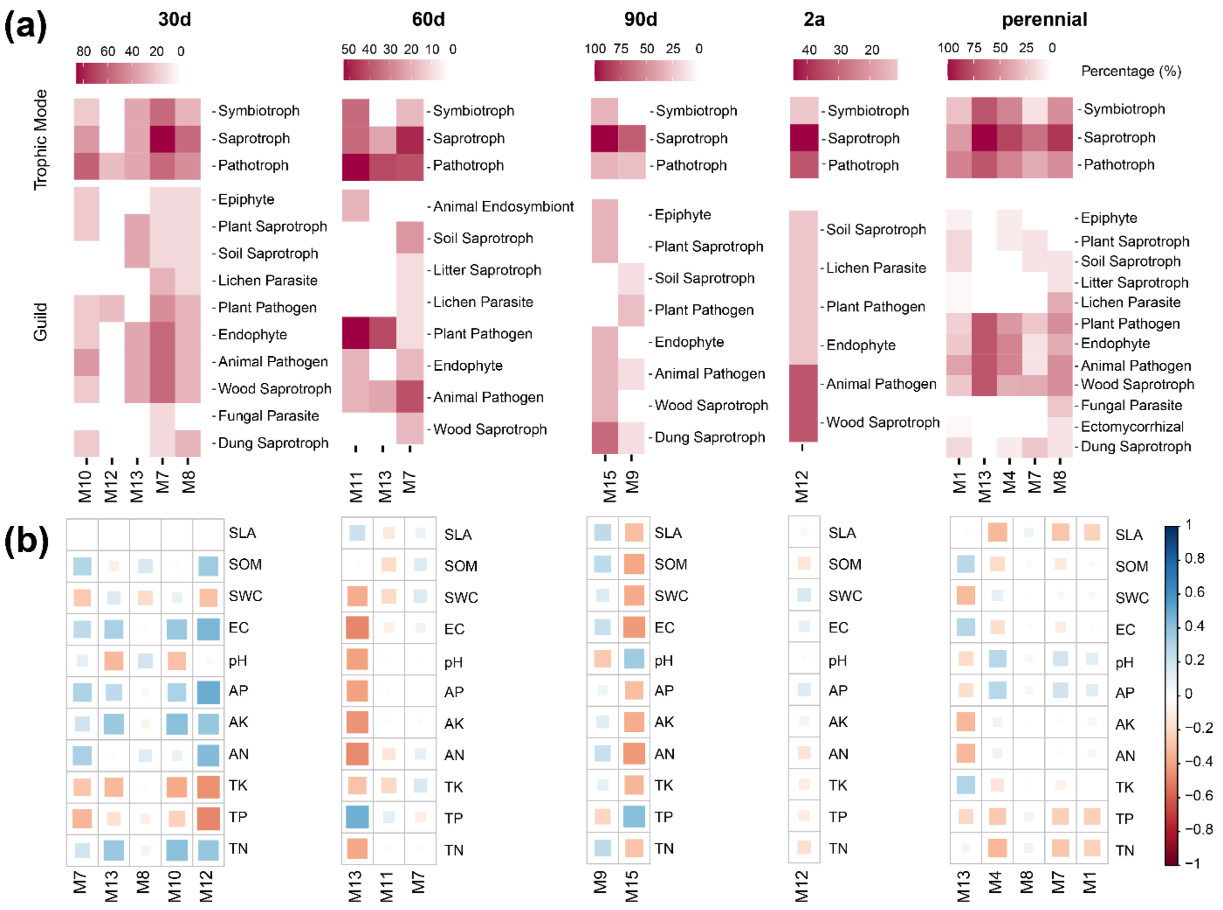

(a)
30d
60d
90d
2a
perennial
Percentage (%)
Trophic Mode
Guild
Symbiotroph
Saprotroph
Pathotroph
Epiphyte
Plant Saprotroph
Soil Saprotroph
Lichen Parasite
Plant Pathogen
Endophyte
Animal Pathogen
Wood Saprotroph
Fungal Parasite
Dung Saprotroph
Animal Endosymbiont
Litter Saprotroph
Ectomycorrhizal
M10
M12
M13
M7
M8
M11
M15
M9
M1
M4
(b)
SLA
SOM
SWC
EC
pH
AP
AK
AN
TK
TP
TN

Figure S6
